# Influence of glutamatergic and GABAergic neurotransmission on obstructive sleep apnea

**DOI:** 10.3389/fnins.2023.1213971

**Published:** 2023-07-13

**Authors:** Piotr Kaczmarski, Marcin Sochal, Dominik Strzelecki, Piotr Białasiewicz, Agata Gabryelska

**Affiliations:** ^1^Department of Sleep Medicine and Metabolic Disorders, Medical University of Łódź, Łódź, Poland; ^2^Department of Affective and Psychotic Disorders, Medical University of Łódź, Łódź, Poland

**Keywords:** obstructive sleep apnea, GABA, glutamate, benzodiazepines, neurotransmission

## Abstract

Glutamate and γ-aminobutyric acid (GABA) are the two main neurotransmitters in the human brain. The balance between their excitatory and inhibitory functions is crucial for maintaining the brain’s physiological functions. Disturbance of glutamatergic or GABAergic neurotransmission leads to serious health problems including neurodegeneration, affective and sleep disorders. Both GABA and glutamate are involved in the control of the sleep–wake cycle. The disturbances in their function may cause sleep and sleep-related disorders. Obstructive sleep apnea (OSA) is the most common sleep respiratory disorder and is characterized by repetitive collapse of the upper airway resulting in intermittent hypoxia and sleep fragmentation. The complex pathophysiology of OSA is the basis of the development of numerous comorbid diseases. There is emerging evidence that GABA and glutamate disturbances may be involved in the pathogenesis of OSA, as well as its comorbidities. Additionally, the GABA/glutamate targeted pharmacotherapy may also influence the course of OSA, which is important in the implementation of wildly used drugs including benzodiazepines, anesthetics, and gabapentinoids. In this review, we summarize current knowledge on the influence of disturbances in glutamatergic and GABAergic neurotransmission on obstructive sleep apnea.

## 1. Introduction

Glutamate is well known for its function as the major excitatory neurotransmitter and can be found in different areas of the human central nervous system (CNS). In a healthy brain, glutamate is responsible for maintaining synaptic plasticity and many physiological functions such as learning and memory consolidation. In pathological conditions, glutamate is described as a neurotoxin leading to neurodegeneration (Niciu et al., 2012).

### 1.1. Glutamate and glutamate receptors

**Figure 1:**
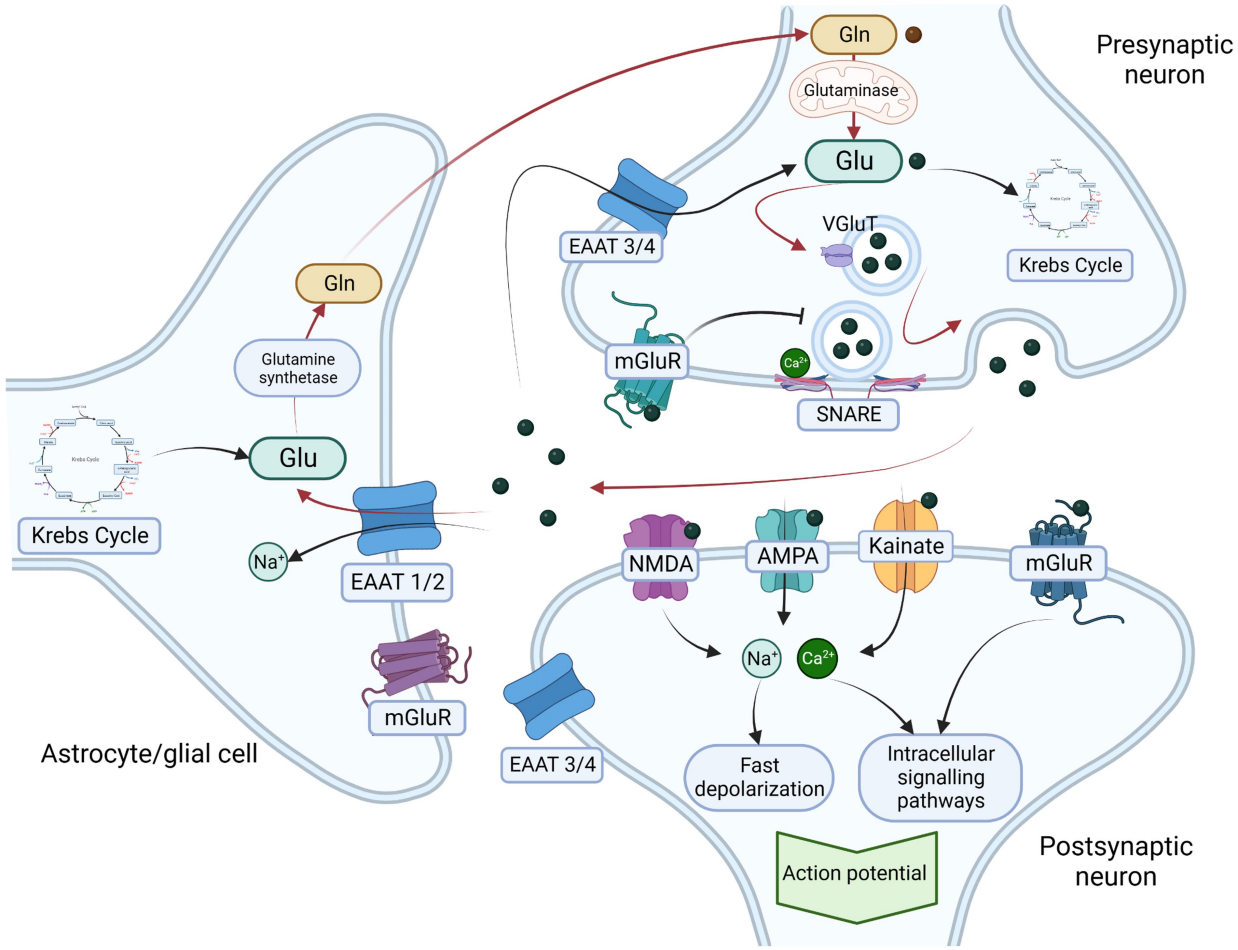
Glutamatergic neurotransmission and glutamate-glutamine cycle. Glutamate is one of the most common excitatory neurotransmitters, It is synthesized *de novo* (from α-ketoglutarate – a product of Krebs cycle) or from glutamine by the mitochondrial enzyme glutaminase. In presynaptic neurons glutamate is transported from cytosol into presynaptic vesicle through glutamate transporter (VGluT). The contents of the vesicle are released in a Ca^2+^ and soluble N-ethylmaleimide-sensitive factor attachment protein receptor (SNARE)-dependent manner into the synaptic cleft. Glutamate receptors include two main groups – ionotropic receptors including N-methyl D-aspartate (NMDA), α-amino-3-hydroxy-5-methyl-4-isoxazolepropionic acid (AMPA), and kainic acid receptor (kainate) and metabotropic glutamate receptors (mGluR). Ionotrpoic receptors act as ion channels that after activation by glutamate induce rapid inflow of sodium (Na^+^) and calcium (Ca^2+^) ions resulting in fast depolarisation of postsynaptic neuron. The metabotropic glutamate receptors (mGluR) are slower acting through membrane bound G-protein and second messenger that results in activation of postsynaptic ion channels as well as intracellular signaling pathways and gene expression regulation. Activation of presynaptic receptors result in glutamate release inhibition. To maintain the excitatory homeostasis glutamaete transoporters regulate its concentration in extracellular matrix, Excitatory amino acid transporters 1 and 2 (EAAT1/2) are mainly localized on actrocytes and other glial cells and respond for transportation of glutamate into the cytosol, where it is metabolized into glutamine by glutamine synthetase. Glutamine is subsequently transported back into presynaptic neurons where it is a substrate for glutamate synthesis. The circulation of glutamate between presynaptic neurons and astrocytes is called glutamate-glutamine cycle (red arrows in the figure) and is one of the most important mechanisms preventing the excitotoxicity. Additionally, excitatory amino acid transporters 3 and 4 that are mainly expressed on neurons also transports excessive glutamate directly into neurocytes. Legend: α-amino-3-hydroxy-5-methyl-4-isoxazolepropionic acid receptor – AMPA; Excitatory amino acid transporters 1 and 2 – EAAT ½; Excitatory amino acid transporters 3 and 4 – EAAT 3 /4; glutamine – Gln; glutamate – Glu; kainic acid receptor – Kainate; metabotropic glutamate receptors – mGluR; N-methyl D-aspartate receptor – NMDA; vesicular glutamate transporter – VgluT. Created with: BioRender.com.

Glutamate is synthesized in glutamatergic neurons *de novo* or it can be recycled in the glutamate/glutamine cycle and it is stored in synaptic vesicles (McKenna, 2007). After the depolarization of the presynaptic membrane, the content of vesicles is released into the synaptic cleft, where it bonds to pre- and postsynaptic receptors. The glutamate receptors are very complex and numerous, they can be divided into ionotropic and metabotropic receptors. The main ionotropic glutamate receptors are *N*-methyl *D*-aspartate (NMDA), α-amino-3-hydroxy-5-methyl-4-isoxazole propionic acid (AMPA), and kainic acid receptor. These receptors after the binding of a ligand, change their conformation allowing for an influx of sodium ions and efflux of potassium ions leading to fast depolarisation of postsynaptic neurons. The metabotropic receptors (mGluRs) can be divided into group I, II, and III, they are slower-acting receptors, that act indirectly on neurons through changes in gene expression and protein synthesis (Niciu et al., 2012). The concentration of extracellular glutamate is tightly regulated to prevent excitotoxicity – the phenomenon of increased glutamate-related overexcitation of neurons that leads to neurotoxicity and degeneration (Dong et al., 2009). It is achieved by glutamate transporters which are located on pre- and postsynaptic neurons as well as on glial cells (mostly astrocytes). The excitatory amino acid transporters (EAATs) are expressed on cells in regions with high glutamate neurotransmission and are responsible for taking up the excess glutamate in the extracellular matrix. Intracellularly glutamate is reversely converted to glutamine by glutamine synthase. The glutamine produced in that way in glial cells is released back to the synaptic cleft where it is taken up by neurons and used in glutamate synthesis. That process is called the glutamate-glutamine cycle and is of great importance for maintaining the homeostasis of the glutamate neurotransmission (McKenna, 2007). Glutamate in the healthy human brain has a few vital functions on a molecular level. It is used in neurocyte cell energy metabolism – intracellularly glutamate via glutamate dehydrogenase can be converted to α-ketoglutarate – a substrate in the Krebs cycle (Andersen et al., 2021). It can be used as an alternative energy source when glucose levels are low. Additionally, glutamate is a key neurogenesis regulator during brain development. It has been described that glutamate receptors are expressed on the surface of neural progenitor cells and that their migration, proliferation, and differentiation may be modulated by the glutamatergic system (Berg et al., 2013; Jansson and Åkerman, 2014). Another role of glutamate is the modulation of synaptic transmission. Metabotropic glutamate receptors may change the excitability of neurons through influence on membrane ion channels activity, especially L-type and N-type voltage-gated Ca + channels (Niswender and Conn, 2010). All of that makes glutamate a key regulator of neural function. The physiological functions of glutamate as well as its involvement in the development in several diseseas are summarized in the Table 1. In general taking the complex roles of glutamatergic signaling in the pathophysiology of numerous neuropsychiatric diseases, it is of great importance to acknowledge the impact of other disorders on changes in the glutamatergic system (Figure 1).

**Table 1:** Physiological functions of GABA and glutamate.

Glutamate	GABA
Physiological function on molecular level	
Major excitatory neurotransmitter Neurocyte Energy metabolism – alternative energy source Neurogenesis regulator Synaptic transmission modulator	Major inhibitory neurotransmitter Modulation of excitatory neurotransmission Timing and synchronization of neuronal signals Neurogenesis regulator Prevents excitotoxicity
Physiological brain functions	
Control of movement in basal glia circuitry Maintaining cognitive functions Sleep control Pain signaling	Sleep control Mood/anxiety control Neurodevelopment Regulation of neuroendocrine system Functions outside central nervous system (e.g., control of gastrointestinal motility, modulation of airway tonus, regulation of insulin, somatostatin and glucagone secretion) (Watanabe et al., 2002)
Dysregulation of GABAergic/glutamatergic system and diseases it evokes	
Neurodegenerative diseases (Alzheimer’s disease, Huntington’s disease, Amyotrophic lateral sclerosis (ALS), Parkinson’s disease) Epilepsy Seizures Depression Schizophrenia Stroke Traumatic brain injury Hepatic encephalopathy (Dávalos et al., 1997; PJ and PG, 1997; Sheldon and Robinson, 2007; Barker-Haliski and White, 2015; Guerriero et al., 2015; Strzelecki et al., 2015; Limón et al., 2021)	Sleep disorders (insomnia, hypersomnia) Neurodevelopmental diseases (autism) Neurodegenerative diseases (Alzheimer’s disease, Huntington’s disease, Parkinson’s disease,) Mood disorders (depression) Addictions Schizophrenia Epilepsy Traumatic brain injury (Hsu et al., 2018)

### 1.2. γ-aminobutyric acid (GABA) and GABA receptors

**Figure 2:**
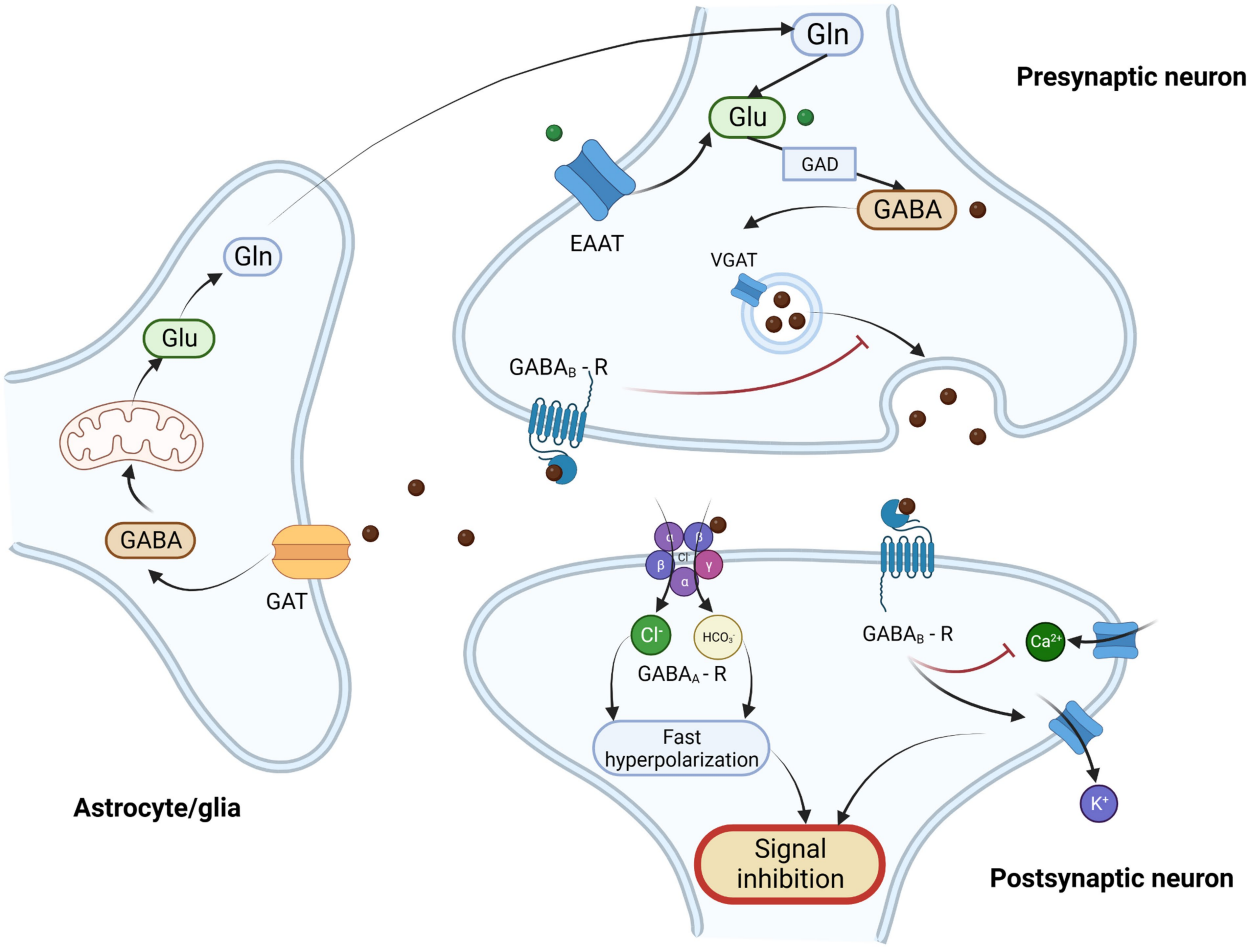
Schematic illustration of GABAergic neurotransmission and GABA/glutamate cycle. γ-Aminobutyric acid (GABA) is the most important inhibitory neurotransmitter. It is synthesized from glutamate (Glu) in presynaptic GABAergic neurons by glutamate decarboxylase (GAD) and next it is transported into synaptic vesicles through vesicular GABA transporter (VGAT). After the depolarization of presynaptic neuron GABA is released into the synaptic clef, where it binds to its post and presynaptic receptors. There are two types of GABA receptors – ionotropic GABA_A_ receptor (GABA_A_-R) and metabotropic GABA_B_ receptor. GABA_A_ receptor is heteropentomer that forms a chloride channel, that after binding of ligand becomes permeable and allows the influx of chloride ions and to a lesser extent carbonate ions, what results in fast hyperpolarization of postsynaptic neuron. GABAB receptor is metabotropic, G-protein coupled receptor, that exerts its action through inhibition of adenylate cyclase, inhibition of calcium channel (Ca2+) and through direct activation of kalium ion channels (K+) resulting in inhibition of neurotransmitter release and modulation of neuronal excitability. Activation of presynaptic GABA receptors results in the inhibition of GABA release from presynaptic neurons. The excessive amount of GABA is taken up from extracellular matrix by GABA transporter (GAT) in astrocytes, and subsequently metabolized through series of enzymatic reaction and Krebs cycle into glutamate and glutamine (Gln). Then glutamine is then transported into the presynaptic neuron where it serves as a substrate for glutamate and GABA synthesis. This process of GAB_A_ recycling is called glutamine-glutamate/GABA cycle. Glutamate may also be transported into presynaptic neurons by excitatory amino acid transporter (EAAT) from the extracellular matrix. Legend: EAAT – excitatory amino acid transporter; GABA - γ-Aminobutyric acid; GABA_A_ – R – GABA_A_ receptor; GABA_B_ -R – GABA_B_ receptor; GAD – glutamate decarboxylase; GAT – GABA transporter; Gln – glutamine; Glu – glutamate; VGAT – vesicular GABA transporter. Created with: BioRender.com.

As mentioned above glutamate as the main excitatory neurotransmitter needs to be regulated and its extracellular concentration must be kept in the strict range, so as not to induce neurodegenerative effects. To maintain the homeostasis of a healthy brain it is important to achieve the delicate balance between glutamate and the main inhibitory neurotransmitter γ-aminobutyric acid (GABA). GABA is synthesized in presynaptic neurons from glutamate, stored in presynaptic vesicles, and released upon membrane depolarization. After releasing to the synaptic cleft, GABA binds to the postsynaptic ionotropic receptor GABA_A_ and pre- and postsynaptic metabotropic receptor GABA_B_ (Ghit et al., 2021). GABA_A_ receptors form a heteropentamer that acts as transmembrane chlorine channels composed of five subunits two α, two β, and one γ subunit (it can also contain δ, ε, θ, π subunits). There are 19 subunit genes that encode six α (alpha1-6), three β (beta1-3), three γ (gamma1-3), three ρ (rho1-3), and one of the δ (delta), ε (epsilon), π (pi), and θ (theta) subunit (Ghit et al., 2021). The GABA_A_ receptor composition is different in specific regions in CNS as well as even within one neuron. The most common isoform of GABA_A_ is composed of α1, β2, and γ2 subunits. The different isoforms of the receptor differ in terms of functional and pharmacological properties. Morphologically GABA_A_ consists of three domains important for its pharmacological targets – an extracellular domain, a transmembrane domain, and an extracellular domain. Receptor activation results in a fast influx of chlorine ions which results in hyperpolarization and functional inhibition of postsynaptic neurons (Ghit et al., 2021). This type of GABA receptor is also a target for a large number of pharmacological agents modulating receptors function including benzodiazepines, barbiturates, ethanol, and general anaesthetics (propofol, etomidate, isoflurane) (Lobo and Harris, 2008; Garcia et al., 2010; Ghit et al., 2021; Goldschen-Ohm, 2022) (see Table 2). GABA_B_ receptors (GABA_B_R) act indirectly on neurotransmission through G-protein and intracellular messengers, and it mediates the slow response of the neuron. The summary of GABAergic neurotransmission is illustrated in Figure 2. GABA may be found throughout the CNS, although it is mainly localized in the interneurons. GABAergic neurons are a part of neural pathways connecting different brain structures forming a neural circuit and, in that way, regulating the activity of these regions (Terunuma, 2018). The physiological functions of GABA as well as its involvement in the pathogenesis in the various diseases is summarized in the Table 1.

**Table 2:** The most common therapeutic agents that act in GABAergic system, and their effects in OSA patients.

Therapeutic agents	Target binding site	Action on GABAergic neurotransmission	Pharmacological effect	Action in OSA and sleep
Positive allosteric modulators of GABA_A_				
Benzodiazepines	GABA_A_ receptor – interface between α and γ subunit in extracellular domain (Luo and Balle, 2022)	Increase the receptor response to endogenous GABA Inhibit transmission of action potential	Sedative Anticonvulsant Amnestic Anaesthesia Myorelaxation Anxiolytic	↑ risk of OSA development ↑ risk of acute respiratory failure ↑risk of comorbid central sleep apnea ↓ nocturnal SaO_2_ nadir ↑ Arousal threshold ↓UA muscle tone ↓ respiratory response to hypoxia and hypercapnia (Drummond, 1996; Gonçalves et al., 2013; Wang et al., 2019)
GABAergic non-benzodiazepine hypnotics	GABA_A_ receptor – interface between α and γ subunit in extracellular domain	Increase the receptor response to endogenous GABA Inhibit transmission of action potential	Sedative Hypnotic	↑ CPAP adherence ↑ arousal threshold ↑ sleep efficiency No change in AHI (slight improvement of AHI by eszopiclone) Little/none effect on UA muscle tone (Rhodes et al., 1990; Eckert et al., 2011; Nigam et al., 2019; Luo and Balle, 2022)
Propofol	GABA_A_ receptor – interface between α and β subunit in transmembrane domain	Agonist potentiation of GABA_A_ receptor; Direct activation of GABA_A_ receptor	Anaesthesia Sedation	↑ risk of respiratory depression ↓ genioglossus muscle activity ↑ upper airway collapsibility ↓upper airway cross-sectional area No change in AHI in postoperative night (Vasu et al., 2012; Ehsan et al., 2016; Fassbender et al., 2018; Shin et al., 2018)
Etomidate	GABA_A_ receptor – interface between α and β subunit in transmembrane domain	Agonist potentiation of GABA_A_ receptor; Direct activation of GABA_A_ receptor	Anaesthesia Sedation	↓ risk of ventilatory depression compared to other iv anaesthetics (Valk and Struys, 2021)
Barbiturates	GABA_A_ receptor – interface between α/β subunit and between β/γ subunit in transmembrane domain	Agonist potentiation of GABA_A_ receptor; Direct activation of GABA_A_ receptor; Block of GABA_A_ chlorine channel – at high concentrations	Anxiolytic Hypnotic Anaesthesia	↑risk of comorbid central sleep apnea ↑ upper airway resistance ↑ time to arousal ↑ genioglossus muscle activity (Takhar and Bishop, 2000; Eikermann et al., 2010; Vega Alanis et al., 2020)
Silent allosteric modulators of GABA_A_				
Flumazenil	GABA_A_ receptor – interface between α and γ subunit in extracellular domain (BZD binding site)	Competitive inhibition of the activity at the BZD binding site on the GABA_A_ receptor; Reversing the effects of benzodiazepines; No influence on the effects of endogenous GABA	Reversal of sedation therapy	No significant changes in PSG features ↓ BZD associated nasal resistance and possibly ↓ upper airway obstruction ↑ flumazenil dose requirement for BZD associated respiratory depression in OSA patients (Schönhofer and Köhler, 1996; Oshima et al., 1999; Seelhammer et al., 2018)
GABA B agonists				
Baclofen	GABA_B_ receptor agonist	Reduces release of excitatory neurotransmitters from presynaptic neurons	Spasmolytic Myorelaxant	↑ total sleep time No clear effect on AHI ↓ respiratory drive (Finnimore et al., 1995)
Sodium oxybate/hydroxybutyrate	GABA_B_ receptor agonist GHB binding site agonist	Reduce the amplitude of excitatory postsynaptic potentials ↓ release of glutamate	Narcolepsy/cataplexy treatment Anesthetic Sedative Hypnotic	↑ risk of respiratory depression Depression of central nervous system ↓ excessive daytime sleepiness ↑ daytime alertness ↓ sleep onset latency ↑ slow wave sleep duration (Pardi and Black, 2006)
GABA derivatives				
GABA-pentoids	α_2_δ subunit-containing voltage-dependent calcium channels (VDCCs)	No direct effect on GABA_A_ or GABA_B_ receptors Increase expression of GAD and therefore increase GABA concentration Modulates the release of excitatory neurotransmitters	Anticonvulsant, Analgesic, Anxiolytic	↑ AHI during non-REM sleep and in the supine position ↑oxygen desaturation index ↑ total sleep time and sleep efficiency ↓ number of aweakings (Cai et al., 2012; Carvalho et al., 2022)

### 1.3. Obstructive sleep apnea

Obstructive sleep apnea (OSA) is a common chronic disease associated with respiratory events during sleep (Friedman et al., 1999). It is characterized by repetitive collapse of the upper airway resulting in intermittent hypoxia and sleep fragmentation (Strollo and Rogers, 2009). OSA is known to promote a number of comorbid disorders including among others: glucose metabolism impairment, cardiovascular diseases (e.g., hypertension), affective disorders, pulmonary diseases, and cognitive impairment (Gosselin et al., 2019; Gabryelska and Białasiewicz, 2020; Gabryelska et al., 2021; Kaczmarski et al., 2022; Karuga et al., 2022). Its high prevalence, association with numerous potentially life-threatening diseases, and severe reduction of patients’ quality of life make OSA one of the most dangerous civilizational diseases of the 21st century. The research of mechanisms underlying OSA that promote its comorbidities has been ongoing in recent years (Gabryelska et al., 2022, 2023; Gabryelska and Sochal, 2022). Glutamate and GABA, two major neurotransmitters in CNS, that regulate many physiological functions including sleep, have been a topic of interest to sleep scientists in terms of their involvement in the complex pathophysiology of the OSA (Macey et al., 2016). In our review, we summarize current knowledge on the dysregulation of glutamatergic and GABAergic systems in OSA and possible therapeutic approaches aiming the dysfunctional neurotransmission.

### 1.4. Central sleep apnea

Discussing the correlation between sleep respiratory disorders and dysregulation in neurotransmitter systems, it is crucial to describe the difference between central and obstructive sleep apnea. In contrast to obstructive sleep apnea, in which the episodes of apnea/hypopnea are caused by collapsed upper airways, although the respiratory effort is present, central sleep apnea (CSA) is characterized by lack of respiratory drive during sleep resulting in repetitive periods of apnea. The pathogenesis of OSA as well as CSA may overlap, and therefore central respiratory events may frequently evoke obstruction in upper airways and obstructive events may result in central respiratory events. Symptoms and complications of both diseases may be also similar including excessive daytime sleepiness and increased risk of adverse cardiovascular outcomes. Nevertheless, there are several pathomechanisms involved in the development of CSA, including treatment related CSA, CSA due to other medical condition (e.g., coronary artery disease), high altitude related CSA and primary CSA. The most important mechanism underlying CSA is unstable ventilatory drive. The proper responsiveness to chemoreceptor stimuli is an important factor for homeostasis of ventilatory drive. Changed chemosensitivity to PaO2 or PaCO2 or H+ concentration could lead to destabilization of respiratory system feedback loop and therefore evoke central respiratory events. Transition from wakefulness to sleep is another factor contributing to the decreased chemosensitivity and can significantly disturb loop gain (Eckert et al., 2007). These are two of many pathophysiological pathways leading to this complex disorder, that could be affected by the changes in neurotransmission in central nervous system. Although CSA is an important sleep respiratory disorder, in this review we aim to focus on the aspect regarding the influence of GABA and glutamate dysregulation on OSA due to its higher prevalence (up to 38% of OSA (Senaratna et al., 2017) vs. 0.9% of CSA (Donovan and Kapur, 2016) in general adult population) and overleaping pathophysiology with CSA.

## 2. Role of GABA and glutamate in physiological sleep/wakefulness regulation

Brain mechanisms involved in the control of sleep and wakefulness are very complex and still not fully known. The regulation of the sleep–wake balance is achieved by the complex interactions between several neurotransmitter systems in different parts of the brain. In recent years many pharmacological agents, that target specific neurotransmitters’ receptors have been designed to induce sleep. That fact highlights the role of neurochemistry in sleep/wake control. Both GABA and glutamate as two major inhibitory and excitatory neurotransmitters in the human brain are the main regulators of wakefulness and sleep.

Wakefulness may be described as a characteristic pattern of behavioral and cognitive responses to the external world (Brown et al., 2012). It is characterized by specific neurological mechanisms of its promotion and control leading to changes in brain activity. Neuronal pathways responsible for maintaining wakefulness create a network of nerve fibers ascending from the brain stem to the cerebral cortex, which is called ascending reticular activating system (ARAS). Multiple neurotransmitter systems contribute to the generation of wakefulness and activation of ARAS. Glutamatergic neurons create thalamocortical projections – a part of ARAS important in the transmission of external sensory information to the cortex and generation of electroencephalographic (EEG) rhythms characteristic of wakefulness (Watson et al., 2010; Brown et al., 2012). Glutamate is also involved in the switch between sleep and conscious state. The role of glutamate in maintaining consciousness has been used in the development of anesthetic agents. Ketamine as an NMDA receptor antagonist is used to inhibit glutamatergic pathways involved in ARAS and therefore induce an anesthetic effect (Brown et al., 2012). Inhibiting GABAergic neurons in association with excitatory glutamatergic neurons in the cortex and subcortical areas is crucial for the generation of cortical low voltage fast frequency EEG rhythms specific for wakefulness. Apart from that in the thalamic reticular nucleus, GABAergic neurons regulate thalamocortical rhythms during sleep/wake transitions (Brown et al., 2012).

Sleep is the state of mind and body characterized by reduced interactions with the surrounding world, subjectively perceived as a loss of consciousness. The changes in brain activity during the onset of sleep reflect in EEG as the replacement of low-amplitude high-frequency rhythms by high-amplitude slow rhythms. This represents the progressive inhibition of ARAS neurons activity during the transition from wake to sleep (Brown et al., 2012). Sleep can be divided into two main phases non-rapid eye movement sleep (NREM) and rapid eye movement sleep (REM) due to the EEG characteristics and brain processes involved in each stage. NREM sleep is composed of 3 stages and is characterized by low skeletal muscle tone and slow eye movement in electrooculography. It starts at the beginning of sleep when the first stage of NREM (N1) occurs immediately after the transition from the wake state. GABA is one of the most important neurotransmitters involved in the sleep/wake switch (Brown et al., 2012). It has been described that the hypothalamus, a main regulator of sleep in the human brain, can be divided into the anterior sleep-promoting area and posterior wake-promoting area. In animal models, a group of sleep-active neurons has been identified in the ventrolateral preoptic nucleus in the hippocampus which mostly consists of neurons containing GABA and galanin that project to wake-promoting nuclei of ARAS. Its activation contributes to the inhibition of ARAS and the promotion of NREM sleep onset. REM sleep in contrast to NREM is characterized by similarity to wakefulness – in terms of EEG rhythms (theta waves, increased cortical activation). REM has its own specific features including muscle atonia and sleep dreaming. GABA has been reported to contribute to muscle atonia during REM sleep. It has been suggested that during REM sleep descending pontine glutamatergic projections excite GABAergic/glycinergic neurons of the bulbar reticular formation, which inhibit spinal motoneurons and result in muscle atonia (Watson et al., 2010; Brown et al., 2012). The above-mentioned mechanisms of sleep regulation are examples of GABA/glutamate involvement in the regulation of sleep/wake control. Dysregulation of GABA neurotransmission has been observed in sleep disorders including narcolepsy and sleep behavioral disorders (Huang and Guilleminault, 2009; Brown et al., 2012).

## 3. Influence of inhibitory/excitatory neurotransmission on OSA

### 3.1. Glutamatergic neurotransmission and OSA

Glutamate, a major excitatory neurotransmitter, is involved in maintaining homeostasis of physiological sleep. As OSA is one of the most common sleep disorders, it has been suggested that dysfunction of the glutamatergic system may be involved in its pathophysiology and lead to the development of OSA comorbidities.

Recent research on animal models has provided evidence concerning the role of glutamate in the development of OSA. The authors of the study observed that injection of L-glutamate into the rat insular cortex resulted in the decrease of genioglossus muscle electrical activity and therefore induced obstructive apnea (Cui et al., 2012). This phenomenon is interesting as the proper genioglossus tonus is important for maintaining the patency of the upper airways. Lowered genioglossus activity due to glutamate stimulation of the insular cortex may lead to upper airways’ collapse – one of the most important pathogenetic factors in OSA. The insular cortex has been described to be involved in respiratory regulation in OSA patients (Macefield et al., 2006). It has been hypothesized that the insular cortex may be injured in OSA (Macey et al., 2008). The mechanisms of this phenomenon are still unknown, although some authors suggest the role of glutamate-mediated cytotoxicity (Macey et al., 2017). What is more in the lateral insular cortex of patients with OSA glutamate/creatine ratio has been found to be significantly upregulated compared to the control group (Kang et al., 2018). Additionally, OSA patients had higher scores on the Hamilton Anxiety Rating Scale and the Hamilton Depression Rating Scale (Kang et al., 2018). The described changes in insular cortex metabolism, especially the increased glutamate levels may also be a reason for neuronal apoptosis. The extensive connections between the insular cortex and the fronto-limbic network involved in the development of depression may lead to a conclusion that possible damage in the insular cortex due to OSA could contribute to the development of affective disorders in OSA patients (Sliz and Hayley, 2012). A similar observation has been made by a group of scientists investigating the changes in the hippocampus. In an animal model study authors evaluated the effect of apnea on hippocampal neurotransmission in guinea pigs (Fung et al., 2007). With the use of electrophysiological studies, they determined that apnea episodes led to an increase of field excitatory potential in cornu ammonis region 1 (CA1) of the hippocampus after stimulation of CA3 in comparison to the control group. Injection of the NMDA receptor antagonist in the CA1 region resulted in a reduction of field excitatory potential observed during apnea episodes (Fung et al., 2007). These results lead to a conclusion that apnea episodes in OSA may abnormally increase the glutamatergic neurotransmission and therefore lead to the apoptosis of CA1 region neurons in the hippocampus via excitotoxicity (Fung et al., 2007). This phenomenon may be a possible pathomechanism of cognitive deficits observed in OSA patients.

The increased glutamate concentration and decreased N-acetylaspartate (NAA, considered as a marker of neuronal viability) have been observed in midbrain nuclei of OSA patients, which indicates neuronal injury. The results of another study show that chronic intermittent hypoxia characteristic of OSA patients decreases glutamate transporters expression in glial cells, which may lead to reduced tolerance to glutamate exposure (Jagadapillai et al., 2014). The excessive amount of free glutamate in the extracellular matrix may lead to excitotoxicity and therefore the neurodegeneration of the area vulnerable to increased concentration of glutamate. In an animal study, it has been observed that an increased concentration of glutamate induced the deterioration of cognitive functions (object recognition, elevated plus maze) (Kumar et al., 2021). The dysregulation of glutamate recycling has been observed in patients suffering from Alzheimer’s disease. In this group reduced expression of glutamate transporter 1 (GlyT1) has been reported, which correlated with the exacerbation of the cognitive dysfunction (Pereira et al., 2016; Andersen et al., 2021; Gasiorowska et al., 2021). Similar excitotoxic conditions prevail during acute ischemic injuries of the brain leading to increased neurodegeneration (Rossi et al., 2000). A recent study on OSA provided evidence, that changes in glutamate transporters plasma concentration in OSA patients are positively correlated with cognitive impairment (Xue et al., 2023). Taking into consideration the novel studies regarding OSA-related cognitive dysfunction the upregulation of glutamate and its excitotoxic effect may be an important pathomechanism underlying this phenomenon (Fung et al., 2007).

Glutamate is often used in research as a metabolite (a molecule representing a biochemical process) of pathomechanism involved in the development of OSA. The recent research on the large cohort database of the Hispanic Community Health Study/Study of Latinos provided evidence that glutamate plasma concentration is positively associated with OSA (Zhang et al., 2022). Glutamate is also often described as a metabolite of a number of OSA adverse health outcomes including adiposity, dyslipidemia, hypertension, incident cardiovascular disease, and glucose metabolism impairment (Zheng et al., 2016; Liu et al., 2019). That observation and the fact that glutamate plasma concentration is correlated with CNS glutamate concentration may lead to a conclusion that glutamate in plasma may be used as a biomarker of OSA and its comorbidities (Alfredsson et al., 1988). This thesis is supported by the results of a recent study that shows a positive correlation between plasma glutamate concentration to increased blood pressure in OSA patients (Boneberg et al., 2021). Additionally, authors have observed that glutamate may be reduced after the implementation of continuous positive airway pressure (CPAP) therapy. The pathomechanisms of this correlation remain unclear, although some authors suggest that hyperglycemia in OSA may be responsible for the downregulation of glutamate transporters (Mysona et al., 2009). Another research group explored the role of glutamate in OSA pathogenesis by studying the expression of metabotropic glutamate receptors in superior cervical ganglion (SCG) in rats – an important blood pressure regulator. The expression of mGluR2/3 detected in SCG has been lowered after the exposure of rats to chronic intermittent hypoxia (Wei et al., 2023). This may lead to a conclusion that changes in the glutamate neurotransmission in SCG due to chronic intermittent hypoxia in OSA may be responsible for an increase in blood pressure. It has been described that on carotid body cells expression of glutamate transporters is increased during hypoxic conditions, which may contribute to the chemoreflex regulation in OSA (Li et al., 2020). As we mentioned previously, glutamate serves as an important regulator of neural homeostasis, in terms of cell energy metabolism and synaptic plasticity.

### 3.2. GABAergic neurotransmission and OSA

Dysregulation of GABA in OSA is often correlated with the upregulation of glutamate and could lead to similar effects as described previously. Even though, the function of these two neurotransmitters is co-dependent, distinct GABAergic dysfunction may be involved in the development of OSA and its comorbidities.

In neuroimaging studies, the assessment of the balance of the inhibitory and excitatory neurotransmission in the dorsolateral prefrontal cortex (DLPFC) in patients with sleep-disordered breathing was performed. The proton magnetic resonance spectroscopy showed that the levels of GABA in DLPFC were negatively correlated with AHI, and positively correlated with minimal oxygen saturation during sleep. The fact of hypoxia-mediated decrease in inhibitory GABA neurotransmission in DLPFC of OSA patients may lead to potential excitotoxicity and cognitive dysfunction (Pereira et al., 2017). Another neuroimaging study using magnetic resonance spectroscopy showed that areas of the anterior insular cortex of OSA patients contain lowered GABA and higher glutamate levels compared to the control (Macey et al., 2016). The complex connections of the insular cortex with other structures of CNS make the insular cortex the integrative center linking inputs from different neuronal systems (Kurth et al., 2010). Insular cortex functions include multimodal sensory processing, autonomic control, emotional control as well as regulation of sympathetic and parasympathetic systems. The altered inhibitory/excitatory balance and enhanced glutamate in the insular cortex may enhance the insular influences over other structures. GABA concentration in the insular region plays an important role in interoceptive processing and responses to interoceptive stimuli. Altered GABA/glutamate levels may influence mood and anxiety disorders. Lowered GABA insular concentration has been associated with anxiety and depression possibly through insular connections with the hippocampus and cingulum (Rosso et al., 2014; Wiebking et al., 2014; Soeiro-de-Souza et al., 2015). The high glutamate in the insular cortex may induce excitotoxic processes, injury, and structural changes in the insular region (Macey et al., 2008; Jagadapillai et al., 2014). The overall changes in the activation and structure of the insula in OSA contribute to impaired autonomic responses – high sympathetic tone (Macey et al., 2012). This phenomenon may constitute possible pathogenesis of hypertension comorbid with OSA.

A recent study evaluated the role of GABAergic neurons in the ventral medulla (VM) and its influence on hypoglossal motor activity. With the use of electrophysiological methods, authors observed the existence of a direct inhibitory neuronal monosynaptic pathway from GABA/glycine neurons in VM to brainstem hypoglossal motoneurons. After the activation of VM GABAergic neurons, the inhibitory effect on tongue electromyographic activity has been observed (Dergacheva et al., 2020). These results are especially valuable for the pathogenesis of REM predominant OSA phenotype, correlated with decreased hypoglossal activity during REM sleep. Previously, sleep phase-related changes in the VM region have been observed. GABAergic neurons of VM have been shown to have increased firing rates during REM sleep (Weber et al., 2015). These results support the hypothesis that REM-related episodes of apnea in OSA may be related to the elevated activation of VM GABAergic neurons in VM and therefore inhibition of hypoglossal motor output and increased upper airway collapsibility.

The importance of GABA neurotransmission in the pathogenesis of sleep apnea is also underlined by the research on polymorphisms of GABA receptors. The study on the correlation of different GABA_B_ receptor 1 gene (GABABR1) polymorphisms and OSA has shown that the Phe658Phe polymorphism is associated with apnea-hypopnea index (AHI) and OSA occurrence (Bayazit et al., 2007). Additionally, the study on the Chinese population has shown that single nucleotide polymorphism rs29230 of the GABABR1 gene has been associated with the risk of OSA. GABA dysregulation has also been recently considered to be the effect of increased proinflammatory mediators in OSA. There are studies suggesting that the increase in circulating interleukin-6 (IL-6) characteristic for OSA is inversely correlated with cognitive performance (Ershler, 1993; Imani et al., 2020). One of the interesting findings is that IL-6 is found to mediate age-related loss of GABAergic interneurons through increased neuronal NADPH-oxidase-derived superoxide production. It may be infrared that elevated peripheral IL-6 levels in OSA may be linked to long-lasting cognitive deficits (Dugan et al., 2009). The implementation of CPAP therapy and possible decrease in IL-6 levels may be beneficial for the group of elderly patients at risk for cognitive losses (Burioka et al., 2009).

Changes in GABA levels in body fluids may be used as an indicator of OSA and its severity. A recent study of patients with coexisting OSA and asthma showed that an overnight increase in urine GABA concentration was associated with aggravation of the OSA (Sheludko et al., 2020). Additionally, GABA was also found to be correlated with snoring duration which may be affected by a GABA-mediated decrease in the hypoglossal motor output (Sheludko et al., 2020). In the population of pediatric OSA patients, the evaluation of morning urine levels showed significantly increased levels of GABA and decreased concentration of taurine. These results may suggest that dysregulation of GABA and decreased taurine levels as a neuroprotective agent are indicators of the neurodegeneration (El Idrissi and Trenkner, 1999). The diagnostic approaches using GABA concentration in body fluids may possibly serve as predictors of OSA as well as cognitive dysfunction, although more data is needed to establish the value of GABA as a marker of neural dysfunction in OSA.

### 3.3. Pharmacotherapy in GABAergic system

The involvement of GABA in OSA pathogenesis has brought some issues when it comes to pharmacological therapy and the use of drugs interacting with GABA receptors (See Table 2). Benzodiazepines, as drugs that are often related to reduced muscle tone, have been suggested to potentially increase AHI and risk of OSA (Sloan and Shapiro, 1993) (See Table 2). This effect may be evoked by various mechanisms. Benzodiazepines bind to the GABA_A_ receptor in the interface between α and γ subunits (different from GABA binding side between α and β subunits) and act as a positive allosteric modulators. Their inhibitory effect on neurotransmission in respiratory regions in CNS is responsible for the reduction of central respiratory drive and depression of chemoreceptor responsiveness to the hypercapnia (Rudolf et al., 1978). They may also stimulate peripheral GABA_A_ receptors resulting in a decrease in the ventilatory muscle tonus (Vozoris, 2014). There have been studies providing evidence that the implementation of triazolam in patients with severe OSA increases arousal threshold and results in the prolongation of apnea events as well as greater desaturations, especially in the group of OSA patients with low baseline hypoxemia (SpO2 < 70%). Pentobarbital, a member of barbiturate group of hypnotic drugs, has been also described to increase the time to arousal and stimulates the genioglossus muscle despite increasing upper airway resistance during sleep (See Table 2). GABAergic non-benzodiazepine hypnotics (Z-drugs) have been suggested to be more suitable for OSA patients as the studies regarding this topic have provided evidence that standard hypnotic doses of non-benzodiazepine drugs do not promote impairment of upper airway muscle activity (Carberry et al., 2017). Additionally it has been described that a standard sedative dose of eszopiclone could increase the respiratory arousal threshold and increase the duration of deeper sleep resulting in improvement in breathing control and reduction in AHI. These effect are especially visible in the group of OSA patients with low arousal threshold at baseline (Eckert et al., 2011). Taking under consideration the effects of these commonly used hypnotic/sedative drugs it is important to properly target the sedative therapy for patients with different underlying pathophysiological mechanism of OSA. The patients with low baseline arousal threshold with a high frequency of arousals may benefit from implementing benzodiazepines/barbiturate or Z-drugs into OSA therapy (Eckert and Younes, 2014; Sands et al., 2018). On another hand, hypnotic therapy in patients with high arousal threshold and more profound hypoxemia may evoke the decrease in desaturation and increase in AHI. Additionally usage of hypnotics in combination with CPAP therapy is an important problem in OSA patients. It has been described that short course of eszopiclone during first weeks of CPAP therapy may improve the adherence to CPAP (Lettieri, 2009). It has been suggested that implementation of hypnotics in patients with lower arousal threshold may be helpful in improving CPAP use (Zinchuk et al., 2018). These observations lead to a conclusion that hypnotic drugs need to be cautiously implemented in the therapy of OSA patients. Another group of GABA-related drugs is gabapentinoids – GABA analogs acting on α2δ subunit of voltage-dependent calcium channels, not on GABA receptors. The most frequently used gabapentinoids include gabapentin and pregabalin, common anticonvulsant and analgesic agents. A recent study provided evidence that gabapentin may acutely worsen sleep breathing. The authors of another study through a search of the WHO drug adverse event database assessed that the use of gabapentinoids is related to a significant number of reports of sleep apnea (Revol et al., 2019). Another GABA related drug that has been hypothesized to induce sleep apnea is baclofen, a GABA agonist, and anti-spasmolytic agent used in neurological disorders. In a double-blind, placebo-controlled, cross-over study, researchers evaluated the effect of baclofen on sleep. Baclofen significantly increased total sleep time, and decreased time spent awake, although the association with the respiratory disturbance index has not changed significantly (Finnimore et al., 1995). These results may serve as a warning signal for physicians to correctly adjust therapy for OSA patients as agents modulating the GABAergic system may have several adverse effects in this group.

The use of hypnotic/sedative drugs especially benzodiazepines and barbiturates in patients with coexisting comorbid conditions (e.g., chronic obstructive pulmonary disease or cardiac failure) may induce the central sleep apnea. Although hypnotic drugs may reduce sleep fragmentation, and improve sleep continuity, its depressant effect on the central nervous system may lead to the depressive effect on respiratory centre (Guilleminault, 1990). On the other hand it has been described that use of benzodiazepines in the patients with idiopathic central sleep apnea, may reduce the number of apnea/hyponea episodes (Bonnet et al., 1990). There has been supportive data regarding use of non-benzodiazepine hypnotics in patients with idiopathic CSA. In an open abel trial study it has been found that CSA patients treated with zolpidem have experienced a decrease in number of central hypopneas/apneas and improved sleep continuity (Quadri et al., 2009). These data suggest that sedative agents in CSA apart from its depressant function on respiratory regulation, in certain groups of patients may have a positive effect. Although the large heterogeneity and complex pathophysiology of CSA makes it hard to unequivocally asses the function of hypnotic agents in CSA patients.

Additionally what is worth mentioning is an influence of hypnotic drugs on sleep microstructure. Especially the agonistic GABA A receptor modulators (barbiturates, benzodiazepines, non-benzodiazepine hypnotic agents) even tough different action on GABA A receptor, they produce their hypnotic effect through similar changes in the sleep microstructure. This group of drugs enhance the effect of GABA on GABA A receptors. The main effects of these hypnotic agents on sleep include the increased ability to fall asleep, increased sleep continuity, increase in NREM sleep time and stimulation of spindles appearance in NREM sleep. It has been also described that they may decrease the REM sleep time due to the suppression of REM sleep episodes (Lancel, 1999). Accordingly, GABA A receptor modulators are thought to play an important role in NREM sleep induction and consolidation through attenuation of low frequency components of NREM sleep and stimulation the mechanisms responsible for spindles generation. It has been described that administration of midazolam induced suppression of slow-wave activity within NREM sleep and an increase in spindle activity (Lancel et al., 1996). What is more, other benzodiazepines also showed an effect of increasing total sleep time dose dependently, reducing awakenings and promotion of stage 2 sleep with subsequent reduction in stage 1 sleep, slow wave sleep and REM sleep. Non-benzodiazepine drugs also shorten sleep-onset latency, increase NREM sleep and could decrease REM sleep.

### 3.4. Pharmacotherapy in glutamatergic system

The role of glutamate in OSA pathophysiology has been used to test possible therapeutic agents. Sabeluzole – a glutamate antagonist was implemented in the therapy of patients with OSA. It was found that sabeluzole was responsible for a significant reduction of the oxygen desaturation index in patients with OSA, although the affective effect of this treatment has not been found significant (Hedner et al., 1996). With regard to excitotoxic role of upregulation of glutmate level in OSA, it has been also reported that ceftriaxone – a beta-lactam antibiotic – increases the expression of glutamate transporters in rat models, and therefore modulates the excitotoxic effects of glutamate (Lee et al., 2008). Another proposed pharmacotherapeutic agent that could possibly alleviate the neurotoxic effect of glutamate is eszopiclone – a positive modulator of the GABA_A_ receptor. The results of a study on the effects of eszopiclone on the apnea induced neurotoxicity in guinea pigs provided evidence that experimental recurrent apnea resulted in significant morphological apoptosis of the hippocampal region as well as an increase in synaptic responsiveness (Fung et al., 2009). In the study group admission of eszopiclone suppressed the apnea-induced hyperreactivity and prevented the neurodegeneration of the hippocampal region (Fung et al., 2009). These approaches could serve as new therapeutic agents for OSA in the prevention of the neurotoxic condition, although there is still little evidence on the effect of glutamate-targeted therapies in OSA patients. Apart from possible therapeutic agents for OSA, it is also important to discuss the influence anaesthetic agents commonly used in the daily practice on the course of OSA. It has been described that ketamine, a noncompetitive NMDA receptor antagonist an a potent analgesic and hypnotic agent may abolish the sleep induced upper airway dilatator muscle dysfunction and act as respiratory stimulant (Eikermann et al., 2012). It is especially important for OSA patients undergoing general anasthesia as other anaesthetic drugs including propofol may increase the upper airways collapsibility and induce the perioperative breathing instability what may constitute a serious complications in this group of patients (See Table 2). These observations in connection with the previously mentioned role of glutamate in the development of various OSA comorbidities underlines the importance of glutamatergic neurotransmission in the pathogenesis of OSA and opens new opportunities to investigate new therapeutic approaches for OSA.

## 4. Conclusion

This review summarized the current knowledge on the role of GABAergic and glutamatergic systems in OSA. Available data suggest that dysfunctional neurotransmitter systems might be involved in the pathogenesis of OSA as well as its comorbidities. As GABA and glutamate may have a different effects on the activation of targeted cells, they cooperate to maintain the homeostasis of physiological neurotransmission and structural unity of the brain. The excitatory effect of increased glutamate in different brain areas including the insular cortex and hippocampus may lead to its dysfunction and damage. Excitotoxicity is described as one of the most important mechanisms involved in brain structural changes. Additionally, the increased glutamate concentration in body fluids may serve as a molecular metabolite of OSA pathogenesis. The effects of GABAergic dysregulation in OSA are partially caused by the increase in the excitatory influences leading to similar damage to neurons. GABA is an important transmitter in the regulation of upper airways muscle tonus, which especially manifests during REM sleep and dysregulation of the GABAergic system may manifest as REM-predominant OSA. Overall, both GABA and glutamate may possibly contribute to the development of common OSA comorbidities including affective disorders, cognitive impairment, hypertension, autonomic dysregulation, and dysfunctional glucose metabolism. The functions of GABAergic pathways in OSA pathogenesis are important when assessing the adverse effects of GABA-modulating therapies including gabapentinoids, benzodiazepine, and non-benzodiazepine hypnotics, groups of drugs commonly used to treat OSA patients. The further research is needed for better recognition of complex neurological pathways involved in OSA-related changes in GABA/glutamate balance, as well as to establish possible therapeutic approaches for OSA comorbidities related to dysfunctional neurotransmission.

## Author contributions

AG and PK: creation of article concept. PK: conduction of preliminary literature search, writing original draft, and creation of figures. AG: supervision. AG, MS, DS, and PB: revision of manuscript. All authors contributed to the article and approved the submitted version.

## Conflict of interest

The authors declare that the research was conducted in the absence of any commercial or financial relationships that could be construed as a potential conflict of interest.

## Publisher’s note

All claims expressed in this article are solely those of the authors and do not necessarily represent those of their affiliated organizations, or those of the publisher, the editors and the reviewers. Any product that may be evaluated in this article, or claim that may be made by its manufacturer, is not guaranteed or endorsed by the publisher.
